# Supplementary material for: Direct visualization of charge transport in suspended (or free-standing) DNA strands by low-energy electron microscopy
Source: Sci Rep. 2019 Jun 20;9:8889. doi: 10.1038/s41598-019-45351-4 (PMC6586886; doi:10.1038/s41598-019-45351-4)
Supplement: Supplementary file 3 — Supplementary Information [file 41598_2019_45351_MOESM3_ESM.docx]

Supplementary Information for

Direct visualization of charge transport in suspended (or free-standing) DNA strands by low-energy electron microscopy

Tatiana Latychevskaia^1^, Conrad Escher^1^, William Andregg^2^, Michael Andregg^2^ and Hans-Werner Fink^1^

^1^Physics Department, University of Zurich, Winterthurerstrasse 190, 8057 Zurich, Switzerland

^2^Halcyon Molecular, 505 Penobscot Drive, Redwood City, CA 94063, USA

**Electron dose and radiation damage**

For an electric current of 200 nA, there are 1.248 × 10^12^ electrons flowing per second. Assuming that all these electrons illuminate an area of 1 × 1 um^2^, we obtain 1.248 × 10^4^ electrons per second per 1 Å^2^. This gives 250 electrons per 1 Å^2^ for a 20 ms single hologram frame. For a typical electron dose of 1 × 10^4^ per 1 Å^2^ (1×10^6^ per 1 nm^2^) deposited by 100 eV electrons per second, the radiation dose amounts to^1^:

$$R=\frac{\text{(dose in }e\text{/nm}^{2}\text{)×(energy in eV)}}{\text{(penetration depth in mm)×(density in g/cm}^{3}\text{)}}\cdot0.16=\frac{1\cdot10^{6}\cdot100}{5\cdot10^{\text{-7}}\cdot1.4}\cdot0.16=2.29\cdot10^{13}\text{ Gray,}$$

where we assumed the penetration depth of 5 Å and a typical density of biological specimen of 1.4 g/cm^3^. This thus amounts to 4.58 × 10^11^ Gray per one hologram. Taking into account that at the modest, the resolution of about 1 nm can be achieved when imaging with 100 eV low-energy electrons, the radiation dose exceeds the maximum tolerable dose for high-energy electrons and X-ray by about a factor of 10^4^, as illustrated in Fig. S1. It has been demonstrated that DNA molecules do not exhibit significant radiation damage, at the resolution of 1 nm, when continuously exposed to low-energy electrons for 70 min^2^, thus resulting in a total dose of 9.62 × 10^16^ Gray.


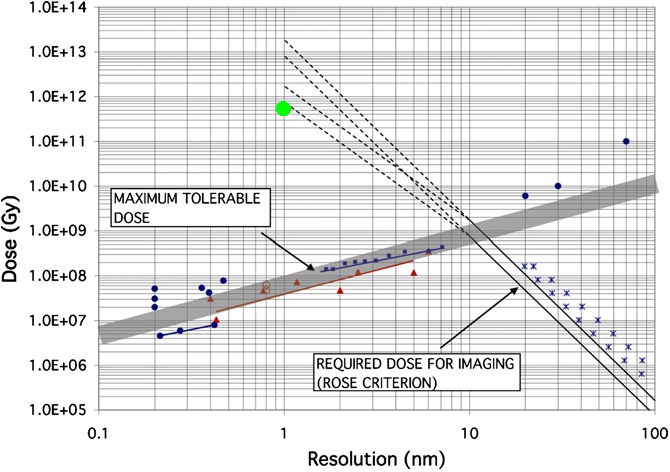


Fig. S1. Graph summarizing information on the required dose for imaging and the maximum tolerable dose. Reprinted from Journal of Electron Spectroscopy and Related Phenomena, Volume 170, Issues 1-3, M.R. Howells, T. Beetz, H.N. Chapman, C. Cui, J.M. Holton, C.J. Jacobsen, J. Kirz, E. Lima, S. Marchesini, H. Miao D. Sayre, D.A. Shapiro, J.C.H. Spence, D. Starodub, “An assessment of the resolution limitation due to radiation-damage in X-ray diffraction microscopy”, Pages 4-12, Copyright (2019), with permission from Elsevier. The green dot indicates the radiation dose for low-energy electrons when imaging biological specimen for 20 ms (one hologram) at 1 nm resolution. The rest of the graph is described in as follows^3^. The types of data from the literature are identified by the symbols as follows: filled circles: X-ray crystallography; filled triangles: electron crystallography; open circles: single-particle reconstruction; open triangles: electron tomography; diamonds: soft X-ray microscopy^3^. The required dose for imaging is calculated for a protein of the empirical formula H_50_C_30_N_9_O_10_S_1_ and a density of 1.35 g/cm^3^ against a background of water for X-ray energies of 1 keV (lower continuous line) and 10 keV (upper continuous line)^3^. The dashed continuations of these lines refer to the transition region from a coherent to an incoherent behaviour. Measurements of the required dose for X-ray imaging are plotted as crosses^3^. The maximum tolerable dose is obtained from a variety of experiments by Howells et al^3^.

**Low-energy electron imaging at different magnification**

Figure S2 shows three images of the ssDNA sample acquired in the low-energy electron microscope at different magnification. The magnification is changed by varying the distance between the electron source and the sample.


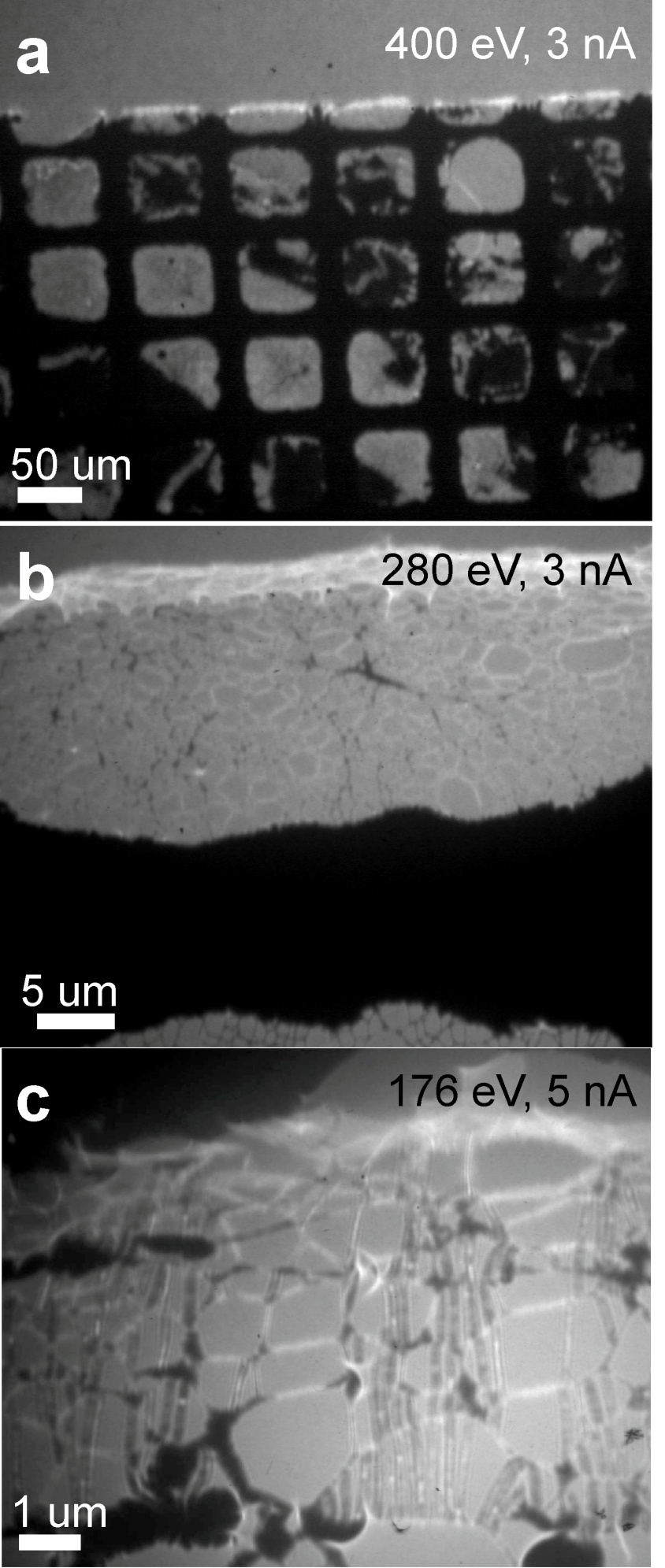


Fig. S2. Images of the ssDNA sample acquired in the low-energy electron microscope at different magnification.

**References**

1 Henderson, R. Cryoprotection of protein crystals against radiation-damage in electron and x-ray dffraction. *Proc. R. Soc. B* **241**, 6–8 (1990).

2 Germann, M., Latychevskaia, T., Escher, C. & Fink, H.-W. Nondestructive imaging of individual biomolecules. *Phys. Rev. Lett.* **104**, 095501 (2010).

3 Howells, M. R. *et al.* An assessment of the resolution limitation due to radiation-damage in x-ray diffraction microscopy. *J. Electron Spectrosc.* **170**, 4–12 (2009).
